# Optimization of tray inventory levels in hospitals from an integral perspective

**DOI:** 10.1007/s10729-025-09743-5

**Published:** 2026-02-19

**Authors:** Hayo Bos, Gaspard Hosteins, Wick Wijnholds, Aleida Braaksma, Gréanne Leeftink

**Affiliations:** 1 Diakonessenhuis Utrecht, P.O. Box 80250, Utrecht, 3508 TG The Netherlands; 2 Management Science, Technical University of Denmark, Akademivej 358, Kongens Lyngby, 2800 Denmark; 3 Center for Healthcare Operations Improvement and Research, University of Twente, P.O. Box 217, Enschede, 7500 AE The Netherlands; 4 Stochastic Operations Research, University of Twente, P.O. Box 217, Enschede, 7500 AE The Netherlands

**Keywords:** Integral planning, Hospital sterilisation, Simulation, Healthcare management, RMD cycle, Tray inventory management

## Abstract

Surgical instrument trays are crucial for any hospital’s surgical department; patients cannot undergo surgery if the right instruments are not available. It is therefore of great significance to provide a steady and sufficient supply of sterile medical devices to the surgery department, which can be guaranteed by having sufficient inventory of sterile trays. However, the value of surgical tray inventory is substantial, and given the pressure on the health care industry to reduce cost, a properly aligned tray inventory is of utmost importance. Choosing the appropriate tray inventory levels is hard due to the complex interplay between the surgical schedule and the sterilisation cycle. In this paper, we propose a practice-driven heuristic and a Discrete Event Simulation (DES) model to choose and evaluate the appropriate tray levels respectively. We propose a novel surgery schedule generation algorithm to account for the dependency in tray demand, which has not been addressed in literature thus far. Using a case study from our partnering hospital, we show that without compromising quality, the tray inventory can safely be reduced by 28%.

## Highlights

- Our study focuses on optimizing stock levels of surgical instrument trays.
- We propose a new heuristic, which we benchmark against the state of the art.
- We develop a Discrete Event Simulation model of the Central Sterilisation Services department of our partnering hospital.
- We propose a new surgery generation algorithm to account for the variability in the demand for surgical instruments.
- We show that we can reduce tray stock levels by 28% without increasing cancellations or rescheduled surgeries.

## Introduction

Sterilisation services are a key part of hospital operations [1]. All surgeries require trays consisting of a fixed configuration of Reusable Medical Devices (sRMDs), which after usage, have to undergo sterilisation before they can be used again. Without ample tray inventory and a well-organised sterilisation process, the surgical schedule may face disruptions or even cancellations: inefficient sterilisation processes may disrupt up to 12% of surgeries due to the lack of supply of surgical tools [2]. Due to ageing populations and budget constraints, hospitals are under increasing pressure to accomplish more with fewer resources. According to Van De Klundert et al. [3], the value of the sterile equipment inventory of hospitals is substantial. Having overly conservative stock levels could therefore heavily impact the hospital’s budget. On the other hand, Viderman et al. [4] show that not having the appropriate equipment available also leads to, among others, waste of resources, financial burden and patient dissatisfaction.

Our partnering hospital is a medium-sized Dutch hospital which uses stock levels which are historically determined, mostly based on (educated) guesses by doctors and management. The hospital aims to expand to face the growing demand for healthcare in the Netherlands. As surgery trays are a vital resource and a hard requirement to facilitate such growth, this motivated us to study the tray stock levels; next to the strong anecdotal evidence that the current levels were suboptimal. During site visits we observed many tray types of which trays had been untouched for a long time, while there was an acute need of other tray types due to insufficient stock. The goal is therefore to develop a method to determine the right stock levels ensuring availability of surgical equipment while preventing wastage of resources. The underlying complexity of the sterilization cycle and tray demand requires a thorough analysis and the development of novel models.

The organization of sterilization services varies. Sterilization services can be centralized, outsourced, or pooled [5, 6]. The common denominator of the organization of sterilization services is that it constitutes a cyclic system, in which, after usage, the trays of RMDs have to undergo sterilization before they can be used again. Our goal is to optimize tray inventory in the Central Sterilisation Service (CSS) context. There is a vast amount of literature on inventory management, see, e.g., [7], but due to the cyclic, complex nature of the CSS, those methods can not be applied. Besides, the demand for surgical trays is largely dictated by the surgical schedule. Since a single surgery often requires multiple trays of various types, tray demand tends to be interdependent, a phenomenon further amplified by surgeon preferences. In contrast, the literature typically assumes demands to be independent. Hence, there exists a disparity between conventional inventory optimization techniques and the optimization of inventory for surgical trays, as indicated in [1]. This is the gap we aim to bridge in this paper.

The key contribution of our work is a holistic framework to determine and assess resource usage at the CSS. Contrary to, e.g., [2, 8], this work takes into account all the components of the surgical tray cycle, thus beyond the sole analysis of the CSS alone. As part of this framework, we propose a surgical schedule generation algorithm, to model the demand for trays from surgery and the outpatient clinic. To the best of our knowledge, this is the first time such a procedure has been included in a DES model of the tray cycle. An important challenge is to determine the appropriate tray stock levels. We propose a practice driven heuristic, which we validated by a comprehensive DES model of a hospital’s surgical tray flow. We compare our heuristic against far more complicated heuristics from recent literature, and show its superior performance. Concluding, we propose a new simulation-based CSS performance evaluation framework, a surgery generation algorithm and a tray inventory optimization heuristic. We show our heuristic’s superior performance using our holistic simulation framework.

In what follows we review the existing literature in Section 2. In Section 3, we describe a general CSS layout and introduce our DES model. Section 4 introduces our inventory optimization heuristic and two heuristics from literature to benchmark against. In Section 5 we present numerical results obtained from calibrating our DES model and applying the heuristics to a partnering hospital, and in Section 6 we summarize our findings, draw conclusions, and discuss potential future work.

## Literature review

The literature on hospital sterilisation services in Operations Management (OM) and Operations Research (OR) is broadly classified into three categories: tray design, flow analysis/optimisation and inventory management.

A large part of the literature dedicated to the sterilisation department focuses on the design of trays, i.e., the blueprint telling which and how many RMDs are in a tray of a certain type. Cardoen et al. [9] proposed that the grouping of RMDs in trays could be formulated as an NP-hard set-covering problem, and various cost-optimisation-based packing strategies have been proposed [9–12]. Moreover, Dobson et al. [10] noted that the optimal design of trays depends on the surgical schedule. Reymondon et al. [13] studied the delayed differentiation of trays. Tray inventory decisions are considered in Dobson et al. [10], while most other literature assumes infinite inventory and focuses on the optimal set covering minimising costs [9, 11, 12].

A second stream of research in the field of sterilisation focuses on flow analysis and optimisation. Di Mascolo and Gouin [8] and Lin et al. [14] proposed simulation modelling approaches to reduce the sterilisation time, from tray arrival in the CSS to sterile storage. A key component of those models is how surgical tray demand is addressed. A common approach is to use historical surgical schedules to evaluate the system’s performance [2, 14]. A drawback of this approach is that potential adjustments are only found retrospectively, and more generic models are needed to devise and validate improvements. A step in this direction is set by Di Mascolo and Gouin [8], who modelled the number of surgeries and the number of trays used as Poisson processes. They, however, lacked a clear link between tray demand and surgery type, preventing the proposed model to use information about the surgical schedule to optimise the sterilisation operation. As up to 90% of surgeries are typically scheduled more than a day in advance [13], the surgical schedule can also be used on an operational level to improve operations, as is done in [8, 13, 15, 16]. Rupnik et al. [2] propose to improve the surgery schedule to reduce CSS bottlenecks, tray stock-outs, and subsequently, surgery perturbations.

In terms of inventory management, research has focused on optimising tray stock levels to minimise purchasing and holding costs [17], and the arrival of sterile trays from an inventory management perspective [3]. Using Markov chains, Diamant et al. [18] developed an approach that calculates the service level for different instrument base stocks in an outsourced sterilisation setup, where trays are sent for sterilisation and become available again 2 days later. Other inventory approaches considered service levels and hospital-specific constraints but are not specifically designed for trays and would need to be adapted to take into account the circular nature of the RMD usage [19–21]. To the best of our knowledge, no previous approach has considered the entire tray cycle, specifically the sterilisation process, when determining the appropriate levels of tray inventory.

To the best of our knowledge, there is no prior work that combines the surgical schedule and sterilisation process information to optimise tray inventory. There is, from a practical point of view, need for straightforward methods to find the appropriate inventory levels, taking the system’s complexity into account. Our approach aims to build a generic simulation model for the sterilisation flow. We incorporate the surgical schedule, which altogether allows for generating long runs and different replications, assessing the effect of demand stochasticity and, therefore, generating insights in the effects of different inventory levels. Building on Rupnik et al. [2], our goal is to improve the sterilisation process, but in a more integrated and holistic manner, including the surgeries and the inventory management to the analysis of the CSS.

## Simulation model

This section presents the proposed simulation model. In Section 3.1, we describe the sterilisation process. In Section 3.2, we translate this process into components which can easily be modelled in most modern DES simulation software. Our surgical schedule generating algorithm dictating the flow of the cycle is explained in Section 3.3. Lastly, Section 3.4 introduces the Key Performance Indicators (sKPIs) to measure the system’s performance.

### Process description of the tray cycle

**Fig. 1:**
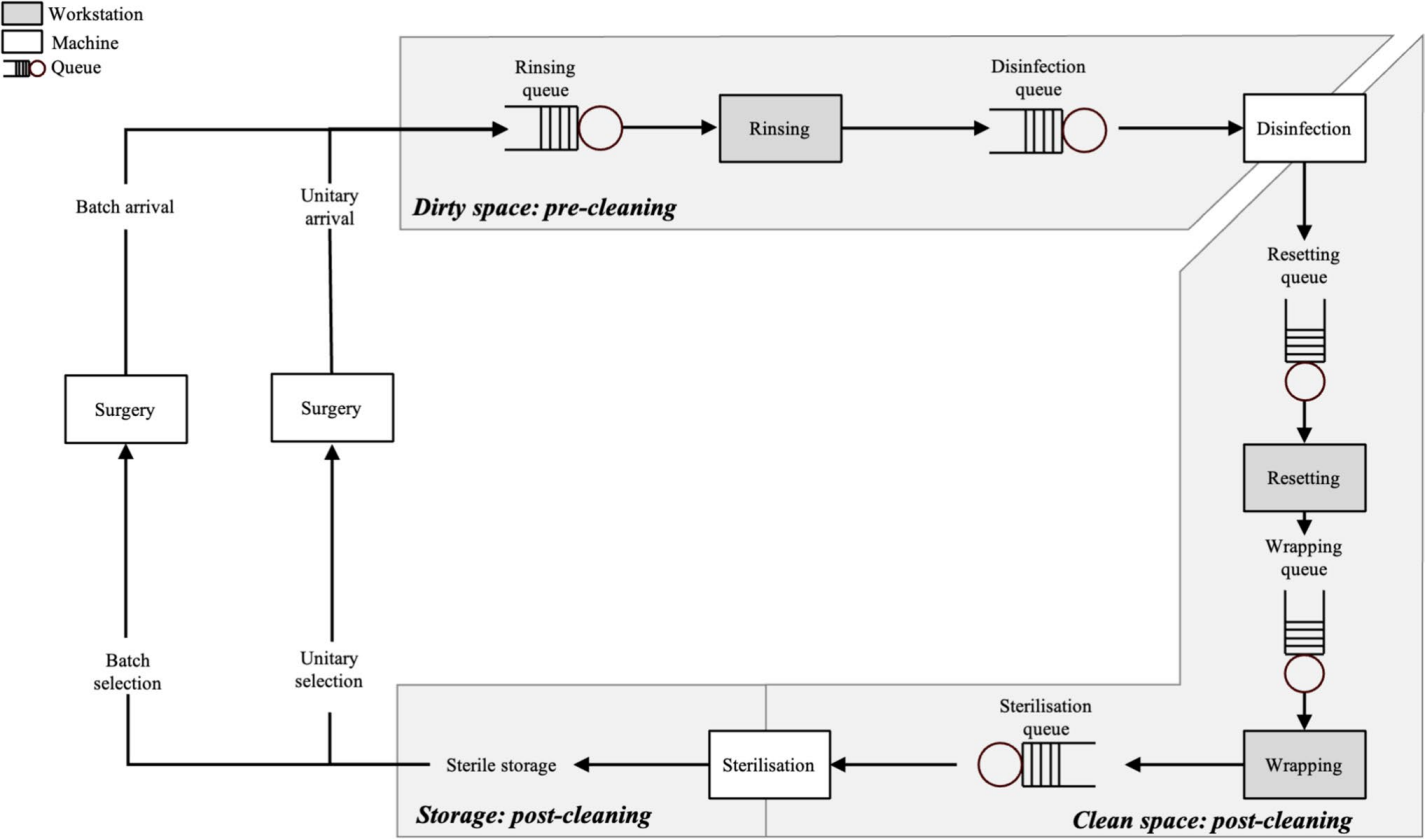
Conceptual model of the sterilisation process

An in-house CSS facility typically consists of three compartments: a decontamination space, a preparation and packing space, and storage [6]. The decontamination space and preparation space are physically separated with trays moving from one to another via disinfection machines. Similarly, the preparation space and storage are physically separated, with trays moving from one to another via autoclaves. The CSS facility is designed such that the risk of contamination is minimised as described by the World Health Organisation report on Decontamination and Reprocessing of Medical Devices for Health-care Facilities [5]. The layout of the CSS is visualised in Fig. 1.

Surgical trays are requested from storage and used for surgical procedures, after which they are sent to the CSS to be cleaned and restocked. In some configurations trays are collected first and sent to the CSS in batches, for instance when used in the outpatient clinic. The trays arrive in the decontamination space. The sterilisation process is composed of several subsequent steps, some of which are manual, others machinated. First, the trays are disassembled into RMDs and pre-cleaned, either manually or automatically, to remove coarse dirt from the RMDs. Following pre-cleaning, the RMDs are loaded and subsequently washed in disinfection machines, after which the trays are unloaded and moved to the preparation space. The RMDs are subsequently manually assembled in their correct tray configurations. The assembly stage includes a check of all tools for maintenance and/or replacement in case of damage, as explained by Fineman and Kapadia [17]. The trays are then manually wrapped in special paper and loaded and sterilised in autoclaves. Afterwards, the trays are restocked in the storage facility.

While the above describes the general layout of CSSs, there frequently are local exceptions. Examples include CSS facilities which also provide sterilisation services to satellite locations or outpatient clinics to which batch transportation has to be arranged [22]. As another example, in certain CSS facilities, not all tray types are allowed to be combined in one disinfection machine charge; trays used for eye surgery and for anaesthetic purposes are to be disinfected separately.

### Model components

The resources of the tray cycle consist of surgical trays, machines, workstations, and staff. The tray inventory can be described by a set $\mathcal {S}=\left\{ 1,\dots ,S\right\}$ of tray types, together with a corresponding inventory $n_1,\dots ,n_{S}$, capturing the inventory per type *s*. Typically, hospitals have different sized trays. The spatial dimensions of trays are important at various points in the sterilisation cycle, e.g., in the disinfection machines and autoclaves. To reflect this, we introduce a size parameter $w_s, s=1,\dots ,S$, recording the size of each tray. Trays usually have a fixed size, and hospitals typically use several sizes.

There are three types of workstations, where trays are pre-cleaned, assembled or wrapped. Let $n_{rw}$, $n_{ew}$ and $n_{ww}$ indicate the number of available workstations with tray capacity $c_{rw}, c_{ew}$ and $c_{ww}$, respectively. As these tasks are performed manually, we allow the durations to be uncertain by modelling them using the lognormal distribution, where each workstation type has individual parameters, as is customary in literature [23–25]. The same holds for the loading and unloading processes of the disinfection machines and autoclaves.

There are two types of machines: the disinfection machines and autoclaves, of which there are $n_{di}$ and $n_{au}$ available. The durations of a washing or sterilising charge are pre-set, and can therefore be considered to be deterministic. In front of all workstations and machines, trays are stacked in queues. All these queues operate as First In First Out (FIFO) queues with infinite capacity. Both machines and work stations have a service capacity $c_{di}$ and $c_{au}$ respectively, dictating the maximum number of trays which can simultaneously receive service.

The staff is divided over two teams, one working in the preparation space (pre-cleaning) and the other in the decontamination space (post-cleaning). The CSS staff works in shifts. A shift on day *t* is encoded by $h_{t,i} = (\text {start}_i, \text {end}_i$), which represents a shift that starts at time $\text {start}_i$ and ends at time $\text {end}_i$. The number of employees scheduled on a shift is denoted by $n_{pre, h_{t, i}}$ for pre-cleaning, and $n_{post, h_{t, i}}$ for post-cleaning. In general, all staff members are multi-skilled and can perform every task, but transitioning between both spaces requires changing clothes for hygienic reasons. This is why the number of transitions between both spaces should ideally be minimized. The information in this section is summarised in Table 1. To model the batch arrivals from, e.g., outpatient clinic and potential satellite locations, we use batch queues as depicted in Fig. 1. Between the arrival times of the batches, all trays are collected and queued and inserted into the rinsing queue at the preset batch arrival insertion times $b_1,\dots ,b_B$.

**Table 1:** Required parameters per resource for CSS simulator

Resource						
	Tray types	Inventory	Size			
Trays	$\mathcal {S}=\left\{ 1,\dots ,S\right\}$	$n_{s},s\in \mathcal {S}$	$w_s, s\in \mathcal {S}$			
	**Quantity**	**Distribution**	**Mean**	**Std**	**Queue**	**Capacity**
*Workstations*						
Rinsing	$n_{rw}$	Lognormal	$\mu _{rw}$	$\sigma _{rw}$	FIFO / $\infty$	$c_{rw}$
Resetting	$n_{ew}$	Lognormal	$\mu _{ew}$	$\sigma _{ew}$	FIFO / $\infty$	$c_{ew}$
Wrapping	$n_{ww}$	Lognormal	$\mu _{ww}$	$\sigma _{ww}$	FIFO / $\infty$	$c_{ww}$
*Disinfection*	$n_{di}$				FIFO / $\infty$	$c_{di}$
Charge		Deterministic	$\mu _{dich}$			
Loading		Lognormal	$\mu _{dilo}$	$\sigma _{dilo}$		
Unloading		Lognormal	$\mu _{diun}$	$\sigma _{diun}$		
*Sterilisation*	$n_{au}$				FIFO / $\infty$	$c_{au}$
Charge		Deterministic	$\mu _{auch}$			
Loading		Lognormal	$\mu _{aulo}$	$\sigma _{aulo}$		
*Staff*	**Day**	**Shifts**	**Quantity**			
Pre-cleaning	*t*	$h_{t, i}=(\text {start}_i, \text {end}_i)$	$n_{pre,h_{t, i}}$			
Post-cleaning	*t*	$h_{t, j}=(\text {start}_j, \text {end}_j)$	$n_{post,d,h_{t, j}}$			

### Surgery process

The pace of the tray cycle is dictated by the surgical schedule, as it both determines the tray demand as well as the arrival time of trays at the CSS. In practice, surgery schedules are frequently developed in several steps by different departments, and are subject to many historical and practical constraints. This makes it hard to exactly replicate the generation of the surgery schedule, as there are many, hard-to-model manual steps involved based on the planners’ expertise. We therefore present a historical data driven surgical schedule generation Algorithm 1 below, which we composed based on the current scheduling logic, together with Subject Matter Experts (sSMEs).

**Algorithm 1:**
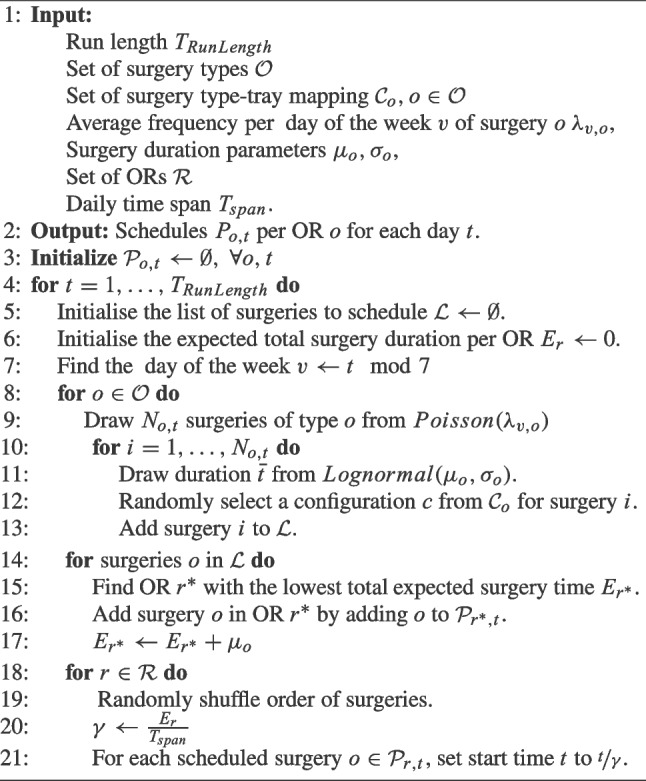
Surgery generation algorithm.

The algorithm requires several inputs including the simulation run length, surgery types $\mathcal {O}$, tray configurations $\mathcal {C}_o, o\in \mathcal {O}$, containing the feasible subsets of trays per surgery type, operating rooms $\mathcal {R}$, surgery durations $(\mu _o, \sigma _o), o\in \mathcal {O}$, and average frequency per surgery per day of the week $\lambda _{v,o}, v\in \left\{ \text {Mon}, \text {Tue},\dots ,\text {Sun}\right\} , o\in \mathcal {O}$, all of which are obtained from the source data. Note that a set of tray configurations $\mathcal {C}_o$ can consist of multiple sets of multiple tray types *s*. The algorithm’s output is a schedule per operating room detailing the surgeries, their start times, and preferred tool configurations.

In Step 3, we initialise the schedule for each operating room *o* and day *t*. We generate a schedule for each day *t* up to the run length. For each day, we initialise the list of surgeries to be scheduled for that day $\mathcal {L}$ as an empty set and set the expected total surgery duration $E_o$ to 0. The day of the week corresponding to *t* is determined in Step 7.

Subsequently, for each surgery type $s\in \mathcal {S}$, we draw the number of surgeries of type *s* to be performed on day *t* in Step 9 using a Poisson distribution, as is done for scheduled arrivals in [26]. The surgery durations are modelled using a lognormal distribution in Step 11, as is common in surgery scheduling literature (see e.g., Zhu et al. [27], May et al. [28], or Schneider et al. [29]). We draw the preferred configuration in Step 12. All surgeries of each surgery type are added to $\mathcal {L}$ in Step 13. Next, we iterate over the list of surgeries to schedule, in each iteration searching for the Operating Room (OR) with the smallest value $E_{r^*}$, i.e., the OR with the most remaining time, in Step 15. Note that this corresponds to a worst-fit heuristic as defined in Shi et al. [30]. Subsequently, we update $E_{r^*}$ in Step 17 by adding the expected duration of surgery *o*. In the last loop, we randomly shuffle the order of surgeries and adjust the starting times of the surgeries such the start times of the surgeries are spread evenly over the day in Step 21. This way, we ensure that tray arrivals are spread over the day, which resembles the actual situation (Table 2).

**Table 2:** Required data for the arrival process parameters of trays

	Data
Surgery types	$\mathcal {O}=\{o_1,\dots ,o_O\}$
Configurations	$\left\{ \mathcal {C}_1,\dots ,\mathcal {C}_O \mid \mathcal {C}_o \subseteq 2^\mathcal {S}\right\}$
Surgery durations	$(\mu _0, \sigma _o), o\in \mathcal {O}$

The DES model executes surgeries per the schedule generated by Algorithm 1. Each surgery is assigned a tray configuration. Surgeries may require multiple trays, and varying configurations of trays may be employed for the same surgery type. By mapping the trays to the surgery demand, we factor in the interdependence of tray demand between those typically used for the same surgeries or scheduled surgeries within the same day. However, if one or more of the trays in the configuration are not at hand, the simulation will iterate over the list of feasible configurations for that surgery, $\mathcal {C}_o$, to check if any other configuration is feasible. If this is the case, the surgery is executed as scheduled with an *alternative configuration*. If this is not the case the simulation will check availability for at most two hours, after which the simulation cancels surgery is cancelled.

### Performance measurement

Our proposed approach focuses on evaluating the tray inventory levels on the performance of the tray cycle within a hospital. The goal is to ensure that surgeries proceed as planned, while having no more trays in stock than necessary. We therefore consider three equally important *performance KPIs*, being the alternative tray percentage, reschedule percentage, and the inventory level.

These are the proportion of surgeries that had to be performed with replacement trays, the proportion of surgeries that are rescheduled due to tray unavailability and the total number of trays in inventory, respectively. The first two are KPIs that reflect the continuity of the operations in the surgical department and are also used by, e.g., [18], and the last KPI reflects on the efficiency of the tray inventory itself.

To validate the simulation model, we additionally consider several *validation KPIs*, which are the average queue lengths before the various workstations and machines, the number of disinfection and sterilisation charges, and the total number of trays cleaned. All KPIs are summarised in Table 3.

**Table 3:** Performance and validation KPIs and their definitions

KPI	Definition
Alternative tray percentage	The proportion of surgeries that had to be performed
	with replacement RMDs.
Reschedule percentage	The proportion of surgeries that are rescheduled due to RMD unavailability.
	A delay in availability of RMD sets of more than 2 hours
	results in a rescheduled surgery.
Total inventory level	The total number of trays in the tray inventory.
Average queue lengths	The average number of trays in the queue before the workstation or machine.
Number of charges	The number of disinfection machine or autoclave charges.
Total number of trays cleaned	The total number of trays that completed the full sterilisation
	cycle during the simulation run.

## Heuristics

To determine appropriate tray stock levels, we propose a straightforward and easy-to-interpret heuristic, which we benchmark against two heuristics from literature. We first introduce our heuristic in Section 4.2, and subsequently summarise the benchmark heuristics presented in [17] in Section 4.3 and the heuristic presented in [18] in Section 4.4. As stated in the introduction, the tray demand is not independent over all trays, as it is driven by the surgery schedule. To circumvent this issue, we use Algorithm 1 to generate the surgical schedule and subsequent tray demand, where we estimate the distribution of the tray demand using its empirical counterpart.

### Notation

Let $Q_s, s\in \mathcal {S}$, denote the total quantity of tray type *s* currently present in the system. With a slight abuse of terminology, we refer to this quantity as the tray inventory level. Note that these trays can be at any of the stages depicted in Fig. 1. Furthermore, let $d_{s,t}$ denote the tray demand for tray type *s* at time *t*. In this section, we propose three heuristics to optimize $Q_s$. We denote the optimized tray level using a superscript corresponding to each heuristic, i.e., $Q_s^H$ is the optimal tray level level for tray type *s* under heuristic *H*.

### Base-stock heuristic

We introduce the Base Stock (BS) heuristic, in which tray type stock levels are set using demand data. This heuristic considers a daily timescale, i.e., we define the tray type demand at day of the week *v*, $d_{v, t}$, to be the number of times a tray from tray type *s* is requested at weekday *v*. Due to the stochasticity of the surgical schedule, $d_{s, v}$ can be modelled as a random variable. Let $F_{d_{s, v}}$ denote its Cumulative Distribution Function (CDF). In the base-stock heuristic, we set the stock level per tray type, $Q_{s}^{BS}(\alpha )$ to the maximum over all weekdays *v* of the $\alpha$-th percentile of the distribution of $d_{s, v}$, i.e.,

1 $$\begin{aligned} Q_{s}^{BS}(\alpha ) = \max _{v} F^{-1}_{d_{s, v}}(\alpha ). \end{aligned}$$

### Fineman and kapadia

Fineman and Kapadia [17] propose a heuristic we refer to as FK, that relates daily tray type demand $\overline{d}_{s}$ with the tray cycle time $\tau _{cycle}$, measured in days. They propose to set the required stock level of tray type $t_i$, $Q_{s}^{FK}$, to

2 $$\begin{aligned} Q^{FK}_{s} = \overline{d}_{s} \cdot \tau _{cycle}. \end{aligned}$$

This heuristic is not directly applicable to our case, as we assume both $d_{s, t}$ and $\tau _{cycle}$ to be stochastic, and $d_{s, t}$ can differ per day *t*. We therefore propose to replace $\tau _{cycle}$ by its expected value $\mathbb {E}(\tau _{cycle})$ and $d_{s, t}$ by its mean, median and max over all days *t*, resulting in $\overline{Q}^{FK}_{s}$, $\widetilde{Q}^{FK}_{s}$ and $\widehat{Q}^{FK}_{s}$ respectively:

3 $$\begin{aligned} \begin{aligned} \overline{Q}^{FK}_{s}&= \left\lceil \mathbb {E}\left( \frac{1}{|\mathcal {T}|}\sum _{t\in \mathcal {T}} d_{s, t}\right) \cdot \mathbb {E}(\tau _{cycle})\right\rceil ,\\ \widetilde{Q}^{FK}_{s}&= \left\lceil \mathbb {E}\left( \text {med}_{t\in \mathcal {T}}\left( d_{s, t}\right) \right) \cdot \mathbb {E}(\tau _{cycle})\right\rceil , \\ \widehat{Q}^{FK}_{s}&= \left\lceil \mathbb {E}\left( \max _{t\in \mathcal {T}} \left( d_{s, t}\right) \right) \cdot \mathbb {E}(\tau _{cycle})\right\rceil , \end{aligned} \end{aligned}$$

where $\text {med} (\cdot )$ denotes the median of its argument, and $\left\lceil \cdot \right\rceil$ is the ceiling function.

### Diamant et al.

Diamant et al. [18] propose a Markov Chain based heuristic to find the appropriate inventory levels, which we refer to as DI. We consider tray type *s*. We discretize the time horizon into time periods of equal length. Let *k* denote the *k*-th period of time. Note that a day *t* may consist of multiple time periods. Diamant et al. [18] assume that inventory can be either sterilised (thus ready-to-use), or dirty, and that at period $k=0$, all inventory is sterilised. They also assume that any trays used in period *k* are sterilised in period $k+1$ and ready to be used again at the start of period $k+2$. Let $Q_s$ be the total amount of inventory of tray type *s* in the system. Similar to the heuristics presented in Sections 4.2 and 4.3, the aim is to find the optimal stock level, $Q_s^{DI}$.

Let $u_{s, k}$ denote the on hand, ready-to-use sterilised inventory of tray type *s* *at the beginning of period k*. Let $v_{s, k}$ denote the number of to-be-sterilised type *s* trays *at the end of period k*. We obtain

4 $$\begin{aligned} \begin{aligned}&u_{s, k} = (u_{s, k-1} - d_{s, k-1})^+ \text { for } k=1,\\&u_{s, k} = (u_{s, k-1} - d_{s, k-1})^+ + v_{s, k-2} \text { for } k=2, 3,\ldots , \end{aligned} \end{aligned}$$

where $(x)^+ = \max {(x, 0)}$. As we can never use more inventory than we have on hand, the ‘dirty’ inventory at the end of the period is $u_{k+1}^s = \min (u_{s, k}, d_{s, k})$. Note that the total inventory is constant, i.e., that $Q_s = u_k^s+ v_{k-1}^s$; inventory is either on-hand or being cleaned. The relation between $u_{s,k}$, $v_{s, k-1}$ and $d_{k-1}$ is as follows:

5 $$\begin{aligned} u_{k, s} = (u_{s, k-1} - d_{s, k-1})^+ + {Q_s}-u_{s, k-1} \\= {\left\{ \begin{array}{ll} Q_s - u_{s, k-1} & \text {if } d_{s, k-1} \ge u_{s, k-1},\\ Q_s - d_{s, k-1} & \text {if } d_{s, k-1} < u_{s, k-1}. \end{array}\right. } \end{aligned}$$

It follows that $$\left\{ u_{s, k}, k = 1, 2,..., K\right\}$$ is a discrete-time Markov Chain, with transition probabilities $$P_{ij}=\mathbb {P}\left( u_{s, k-1}\right.$$$$\left. = i, u_{s, k} = j\right)$$, which depend on the demand distribution. Let $$\left\{ \pi _i(Q_s) = \mathbb {P}(u_{s, k} = i)\right\}$$ denote the steady-state probability distribution of the Markov Chain given a total tray type *s* inventory of $$Q_s$$. Assume i.i.d. demand $$d_{s,k}$$ over all periods *k*. Diamant et al. [18, Lemma 1] provides a closed-form solution for $$\pi _0(Q_s)$$ and $$\pi _{Q_s}(Q_s)$$:

6 $$\begin{aligned} \begin{aligned} \pi _0(Q_s)&= \frac{\mathbb {P}(d_{s, k}=0)\mathbb {P}(d_{s, k}\ge Q_s)}{1 - \mathbb {P}(d_{s, k} \ge Q_s)\mathbb {P}(d_{s, k}\ge 1)},\\ \pi _{Q_s}(Q_s)&= \frac{\mathbb {P}(d_{s, k}=0)}{1 - \mathbb {P}(d_{s, k} \ge Q_s)\mathbb {P}(d_{s, k}\ge 1)}. \end{aligned} \end{aligned}$$

The complete steady state distribution can be obtained by recursion, see [18, Proposition 1]

7 $$\begin{aligned} \begin{aligned} \pi _{Q_s-i}(Q_s)&= \frac{\mathbb {P}(d_{s, k}=i)}{1-\mathbb {P}(d_{s, k}\ge Q_s-i)\mathbb {P}(d_{s, k}\ge i + 1)}\cdot \left( 1-\sum _{j=0}^{i-1}\pi _j(Q_s)\right) \\&+ \frac{\mathbb {P}(d_{s, k}\ge i + 1)\mathbb {P}(d_{s, k}=Q_s-i)}{1-\mathbb {P}(d_{s, k}\ge Q_s - i)\mathbb {P}(d_{s, k}\ge i+1)}\cdot \sum _{j=Q_s+1-i}^{Q_s} \pi _j(Q_s),\\ \pi _{i}(Q_s)&= \frac{\mathbb {P}(d_{s, k}\ge Q_s-i)\mathbb {P}(d_{s, k}=i)}{1-\mathbb {P}(d_{s, k}\ge Q_s-i)\mathbb {P}(d_{s, k}\ge i + 1)} \cdot \left( 1-\sum _{j=0}^{i-1}\pi _j(Q_s)\right) \\&+ \frac{\mathbb {P}(d_{s, k}=Q_s-i)}{1-\mathbb {P}(d_{s, k}\ge Q_s - i)\mathbb {P}(d_{s, k}\ge i+1)} \cdot \sum _{j=Q_s+1-i}^{Q_s} \pi _j(Q_s). \end{aligned} \end{aligned}$$

Let the service level denote the probability that demand does not exceed ready-to-use inventory. For a preset service level $\beta$, the corresponding inventory level $Q_s^{DI}$ is found by solving:

8 $$\begin{aligned} \beta = 1 - \sum _{i=0}^{Q_s^{DI}} \pi _i(Q_s^{DI}) \mathbb {P}(d_{s, k}> i). \end{aligned}$$

## Experiments and computational results

In this section we present the numerical results of our study. Section 5.1 presents the instance based on our partnering hospital. We validate our simulation model in Section 5.2, and apply the proposed heuristics in Section 5.3.

### Case study

This research is conducted at a medium-sized hospital with a main location and a sattelite location in the Netherlands. This hospital has approximately 500 beds, and an in-house CSS at the main location serving both its main and sattelite location. To ensure comprehensive data for this study, an extensive analysis was conducted using the hospital’s Enterprise Resource Planning (ERP) system. We retrieved data of 2021, and after excluding data related to COVID-19 pandemic restrictions in the Netherlands, a total of 28 weeks of data were used. To complement and enhance the data, on-site observations and interviews with the operators involved in the sterilization and tray preparation processes were conducted. This approach allowed for detailed insights into the procedures and activities involved in the process, which may not have been captured in the ERP data. Examples include the duration of rinsing, resetting and wrapping the trays which, and information on the order in which the machines are loaded. We introduce the resources and their parameters in Section 5.1.1, and the surgery process in Section 5.1.2. Section 5.1.3 introduces a clustering of all tray types to facilitate easier analysis.

#### Resource parameters

The hospital has a tray inventory of 2925 trays, distributed over 1213 tray types. The current tray levels are historically determined and necessarily aligned with surgery demand. Together with an SME, we assigned a size to each tray type. The CSS has two pre-cleaning, two resetting and two wrapping workstations. There are four disinfectors available, as well as three autoclaves. The hospital’s disinfection machine loading rack consists of four layers, each with a capacity of 12 size units, where trays of different sizes are placed. Each disinfection machine has a total capacity of 48 size units. In the autoclave, trays are placed in uniformly sized baskets and the autoclaves are filled according to pure FIFO discipline, with each autoclave having a capacity of 30 trays. The staff works in three shifts on weekdays and one on weekends, and is distributed over the pre-cleaning and post-cleaning area. All necessary information regarding the instance parameters is displayed in Table 4.

Although its sterilization process fits within the framework of Section 3, there are local differences. The trays from the hospital’s satellite location are transported to the CSS facility in the main location in batches. Besides, not all tray types are allowed to be combined in one disinfection machine charge; trays used for eye surgery and for anaesthetic purposes are to be disinfected separately. Trays are picked from the queue and added to the disinfection machine until the next tray does not fit, then the operators search for the next tray in the queue that fits, repeating until no more trays can be added. Due to hygiene regulations, certain trays, such as those used for anesthetic purposes, are cleaned separately.

As observed on-site, operators do not always wait for a machine to become completely full before starting it. To maintain continuous flow, they start the machines once a minimum quantity has been reached. To reflect this behavior, we introduce thresholds $\alpha _{dis} = 20\%$ and $\alpha _{ster} = 50\%$ to ensure that a machine is loaded if it is free and there are enough trays to fill it to at least $\alpha$. These thresholds are calibrated to match the weekly number of machine cycles observed on-site. The risk of tray deterioration and contamination is higher before disinfection, thereby increasing the urgency to process them through the disinfection machines. Trays are safer to store after disinfection and prior to sterilization, explaining the difference in threshold levels for disinfection and sterilization.

#### Surgery process parameters

To model the demand for the outpatient clinic, we leveraged a dataset of log records containing tray movements within the system, which consisted of 321761 rows extracted from the hospital’s ERP. A sample snapshot of the dataset is presented in Appendix A. We filtered the dataset to isolate trays designated for the outpatient clinic and modeled the demand for the outpatient clinic using a Poisson process. To model the inflow of surgeries at the hospital, we extracted a surgery log data set of 50,860 rows from the hospital’s ERP. A snapshot of the surgery data set and the retrieved input for our model is available in Appendix B. We utilized the surgery log data set to map surgery types to their corresponding tray configurations. Each configuration was assigned a probability based its occurrence per surgery type in the data.

**Fig. 2:**
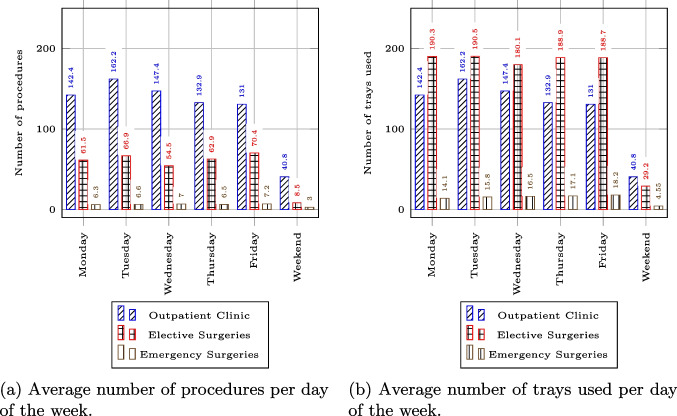
Weekly demand

Figure 2 presents the results of our analysis, displaying the number of procedures occurring on each weekday as well as the corresponding volume of tray use. While the majority of trays are used for surgeries, outpatient clinic procedures are the most frequent, representing over 41.8% of the trays used. Therefore, any comprehensive model of the RMD cycle must account for outpatient demand. Similarly to Reymondon et al. [13], Fig. 2 also reveals that only 10.1% of surgeries are emergencies, indicating significant opportunities for planning and prioritization strategies in the RMD sterilization process.

**Table 4:** Instance parameters based on the partnering hospital

Resource						
	Tray types	Inventory	Size			
*Trays*						
	1213	2925	S,M,L,XL			
	**Quantity**	**Distribution**	**Mean**	**Std**	**Queue**	**Capacity**
*Workstations*						
Pre-cleaning	2	Lognormal	1	1.5	FIFO / $\infty$	1
Resetting	2	Lognormal	2	1	FIFO / $\infty$	1
Wrapping	2	Lognormal	0.5	0.2	FIFO / $\infty$	1
*Disinfectors*	4				FIFO / $\infty$	48
Charge		Deterministic	60			
Loading		Lognormal	2	0.3		
Unloading		Lognormal	0.5	0.2		
*Autoclaves*	3				FIFO / $\infty$	30
Charge		Deterministic	60			
Loading		Lognormal	1	0.3		
	**Day**	**Shifts**	**Quantity**			
*Staff*						
		7.30 AM - 9:45 AM	1			
	Weekdays	9.45 AM - 5 PM	2			
Pre-cleaning		5 PM - 6:30 PM	1			
	Weekends	8 AM - 12:30 PM	1			
		7.30 AM - 9:45 AM	6			
	Weekdays	9.45 AM - 5 PM	9			
Post-cleaning		5 PM - 6:30 PM	3			
	Weekends	8 AM - 1 PM	1			

#### Clustering

To simplify the analysis, we grouped the tray types into three clusters using a constrained K-means algorithm with feature selection, following the algorithm of Wagstaff et al. [31], Bradley et al. [32]. For each tray type, we calculated the expected weekly demand, the number of surgeries and outpatient clinic procedures requiring that tray type, and the current number of trays of that type in the system. The algorithm generated clusters with a minimum size of 25 tray types, ensuring that we obtained meaningful groups of trays for analysis rather than unmanageable outliers or excessively large clusters that a standard classification algorithm might produce.

**Table 5:** Constrained K-means analysis: Tray types clustering

Cluster	Number of	Use	Current stock	Number of	Expected weekly demand
	tray types		per tray type	procedures	per tray type
0	32	Surgeries	9.97	59.94	13.98
1	771	Surgeries	2.15	5.50	0.78
2	410	Outpatient	2.35	1	1.88

Table 5 provides a breakdown of the tray clusters identified through the constrained K-means algorithm. The table shows the number of tray types per cluster, its primary use, the average number of trays in stock per tray type, the average number of procedures for which the tray types in the respective cluster are used, and the expected weekly demand per tray type. Cluster 0 comprises the most frequently used trays in surgeries. These trays have a larger base stock (average of 9.97). Cluster 1 includes all other surgical trays that are less commonly used and more specific to certain types of surgeries. Finally, Cluster 2 consists of all the trays used in outpatient clinics.

### Model validation

We built the DES model in Python (3.10), using SimPy (4.0.1), a process-based discrete-event simulation framework (see SimPy [33], Matloff [34]). The simulation experiments, consisting of 30 replications, a six-week warm-up period and 104 weeks of run length, were run on a Linux cluster with a Xeon Gold 6226R processor and 756GB of memory. Where possible, replications were parallelised across 30 cores to reduce computational time, and each experiment took approximately 50 minutes to run.

To determine the simulation’s warm-up period, we use MSER-m heuristic, introduced by White [35] and White et al. [36] to determine the optimal truncation point for minimising the marginal confidence interval and reducing bias. The MSER-m method uses groups of *m* data points to perform the analysis, increasing its robustness in removing bias [36]. In this analysis, the simulation output data points were already grouped by weeks to account for daily variations, and groups of 2 data points (MSER-2) were used instead of the more common 5 data points (MSER-5), as 5 weeks provided too broad a granularity.

**Table 6:** Warm-up period: Results of the MSER-2 analysis

KPI	Optimal truncation			6 weeks Truncation	
	$d^*$	95% CI Width	in % of mean	95% CI Width	in % of mean
Alternative tray Perc.	4	$\pm 2.67 \cdot 10^{-2}$	$\pm 4.57\%$	$\pm 2.69 \cdot 10^{-2}$	$\pm 4.63\%$
Rescheduling Prob.	2	$\pm 3.56 \cdot 10^{-3}$	$\pm 18.3\%$	$\pm 3.63 \cdot 10^{-3}$	$\pm 18.8\%$
Pre Cleaning queue	2	$\pm 0.265$	$\pm 2.73\%$	$\pm 0.271$	$\pm 2.79\%$
Disinfection queue	2	$\pm 0.129$	$\pm 1.00\%$	$\pm 0.130$	$\pm 1.01\%$
Assembly queue	2	$\pm 9.53 \cdot 10^{-2}$	$\pm 1.62\%$	$\pm 9.76 \cdot 10^{-2}$	$\pm 1.65\%$
Wrapping	2	$\pm 3.47 \cdot 10^{-2}$	$\pm 3.87\%$	$\pm 3.47 \cdot 10^{-2}$	$\pm 3.88\%$
sterilization	2	$\pm 0.339$	$\pm 2.10\%$	$\pm 0.346$	$\pm 2.14\%$

The results of the MSER-2 analysis are presented in Table 6. This experiment consisted of 10 replications and a 3-year (156-week) run length. For each KPI, the table shows the minimal truncation point, which corresponds to the warm-up data that should be excluded from the output analysis to eliminate the initial bias. The alternative tray percentage has the largest truncation point of four weeks, and a conservative approach was taken by using a warm-up period of 6 weeks to remove the initial bias for all KPIs. In addition, Table 6 displays the corresponding marginal 95% confidence interval for each outcome.

**Fig. 3:**
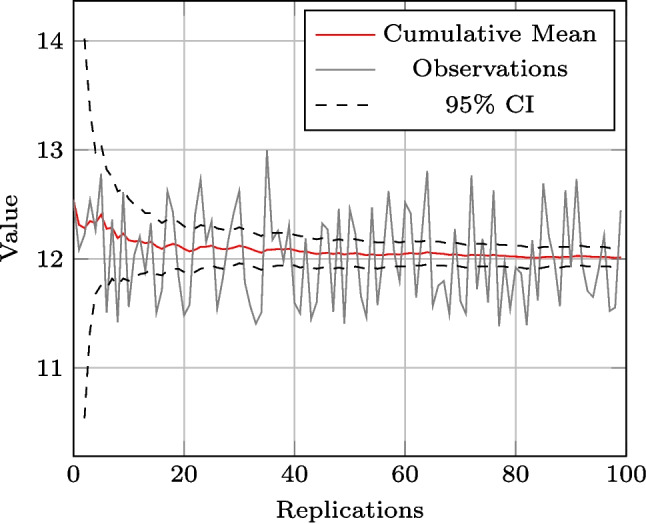
Pre-cleaning queue length cumulative mean and confidence interval by number of replications

To minimise the computational impact of the warm-up period of 6 weeks, a run length of 2 years (104 weeks) added to the 6 weeks of warm-up was used, as recommended by Robinson [37], which reduces the need for replications and therefore warm-up periods. To determine the appropriate number of replications, an experiment of 100 replications was conducted to study the convergence and the width of the 95% confidence interval for the KPIs. As demonstrated by Fig. 3 using the average queue length ahead of pre-cleaning as an example, 30 replications are sufficient to observe convergence of the average values and the 95% confidence interval. This pattern holds for all KPIs, ensuring that 30 replications are adequate to eliminate any bias caused by stochasticity and ensure a steady state.

**Table 7:** 95% confidence interval after 30 replications

KPI	Mean	95% CI Width	in % of mean
Alternative tray Perc.	$4.29 \cdot 10^{-2}$	$\pm 7.08 \cdot 10^{-4}$	$\pm 1.65\%$
Surgery rescheduling Prob.	$1.67 \cdot 10^{-3}$	$\pm 1.34 \cdot 10^{-4}$	$\pm 8.03\%$
Pre cleaning queue	9.07	$\pm 0.154$	$\pm 1.70\%$
Disinfection queue	12.1	$\pm 0.145$	$\pm 1.20\%$
Assembly queue	5.52	$\pm 6.25 \cdot 10^{-2}$	$\pm 1.13\%$
Wrapping	0.804	$\pm 2.10 \cdot 10^{-2}$	$\pm 2.61\%$
sterilization	11.0	$\pm 3.74 \cdot 10^{-2}$	$\pm 0.34\%$

Table 7 displays the 95% confidence intervals obtained from 30 replications. For the majority of the indicators, the confidence intervals are relatively tight and provide accurate results. However, the interval for rescheduling probability is wider. This event is extremely rare with an average of 307.12 occurrences per run (104 weeks). This rarity results in a high volatility in the results and a single event can significantly impact the simulation outcome. We estimate that more than 980 replications would be required to reduce the 95% confidence interval to 5% of the mean value [37]. Although 30 replications may be considered imprecise , it is a trade-off made in consideration of the computational cost that would come with obtaining narrower intervals.

The tray makespan, defined as the time elapsed between tray arrival at the CSS and its placement in sterile storage, is computed by our model. Our model provides a median tray makespan of 3 hours and 18 minutes and a $95^{th}$ percentile of 4 hours and 46 minutes, which have been confirmed by CSS operators and management to closely reflect the actual operations of the CSS.

The CSS does not have precise records of queue lengths or tray-level indicators, so the only available operational data for quantitative validation are the number of trays cleaned per week, and the number of cycles of the autoclaves and disinfection machines. To assess the accuracy of our model, we followed the method proposed by Robinson [38] and calculated the 95% confidence interval of the difference between the means of the simulated output and the operational data extracted from the ERP. The results are presented in Table 8 and show that the confidence interval includes 0, indicating that we do not have sufficient evidence to conclude a significant difference between the simulated output and the operational values.

**Table 8:** Means difference analysis between operational data and simulated output

Metric per week	Op. Data	Simulated	Means difference 95% CI	
	Mean	Mean	Value	% of Op. Mean
Trays cleaned	1770.76	1772.66	$[-46.6,\ +50.4]$	$[-2.63\%,\ +2.85\%]$
Disinfection cycles	172.76	172.61	$[-4.40,\ +4.08]$	$[-2.55\%,\ +2.36\%]$
sterilization cycles	99.79	99.14	$[-2.03,\ +0.72]$	$[-2.03\%,\ +0.72\%]$

### Tray inventory optimization

This section compares the different tray inventory optimization heuristics as introduced in Section 4. For all heuristics, we used daily demand data obtained from the simulation model. We evaluated the BS heuristic by evaluating Eq. 1 for each tray type for $\alpha \in \left\{ 0.60, 0.65,\dots ,0.95\right\}$, which we denote as $BS60, BS65,\dots$. We evaluated Eq. 3 for the average, median and maximum demand to calculate the stock levels for the FK heuristic, denoted by FKmean, FKmed and FKmax respectively. For all three, we estimated the expectation using the sample mean obtained from simulation. Lastly, we evaluated the DI heuristic using Eq. 8 with an initial period length of 4 hours, for $\beta \in \{0.96, 0.97, 0.98, 0.99, 0.995, 0.999, 0.9995, 0.9999\}$, which we denote as $DI96, DI97,\dots$. The results are given in Table 9.

**Table 9:** Numerical results for heuristics

$\alpha$	Total	Reschedule rate			Alternative tray perc		
	inventory						
Current	2925	0.04	$\pm$	0.001	1.20	±	0.01
BS60	1704	0.15	±	0.002	4.72	±	0.03
BS65	1757	0.11	±	0.002	3.98	±	0.02
BS70	1824	0.08	±	0.002	3.03	±	0.02
BS75	1898	0.06	±	0.001	2.44	±	0.04
BS80	1986	0.05	±	0.001	1.94	±	0.01
BS85	2092	0.03	±	0.001	1.38	±	0.01
BS90	2273	0.03	±	0.001	0.87	±	0.01
BS95	2713	0.01	±	0.001	0.38	±	0.01
FKmed	1217	2.07	±	0.01	45.64	±	0.03
FKmean	1216	2.05	±	0.003	45.44	±	0.03
FKmax	1228	1.86	±	0.01	40.50	±	0.02
DI96	1453	0.32	±	0.002	11.47	±	0.05
DI97	1480	0.30	±	0.001	10.47	±	0.03
DI98	1549	0.25	±	0.002	7.97	±	0.02
DI99	1652	0.16	±	0.001	5.79	±	0.02
DI99.5	1774	0.09	±	0.001	3.64	±	0.04
DI99.9	2086	0.03	±	0.001	1.46	±	0.02
DI99.95	2243	0.02	±	0.0004	0.88	±	0.01
DI99.99	2756	0.004	±	0.0004	0.38	±	0.01

As can be seen in Table 9, the BS heuristic performs significantly better than the current inventory policy. For example, for $\alpha =0.85$, the reschedule rate is lower than the current level, while operating on approximately 28% less inventory. For the FK heuristic, there is little difference between the FKmed and FKmean instances, as one might have expected. Interestingly, the FKmax heuristic performs much better with only just over ten trays more than FKmed and FKmean. The DI heuristic performs very well. Compared to the current situation, DI99.9 has slightly less reschedules, but 28.68% less inventory.

**Fig. 4:**
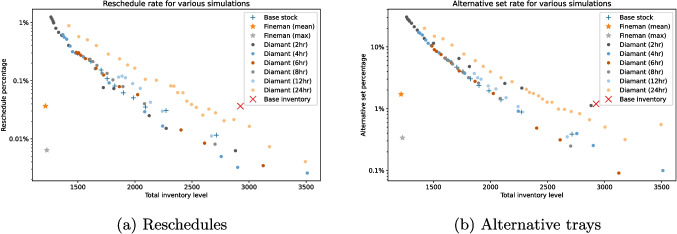
Overview of selected simulation results

As we touched upon before, the choice of period is quite a critical one when using the Diamant et al. heuristic. An overview of selected simulation results can be found in Fig. 4, where we display the total inventory level obtained from the BS, FK and DI heuristics, the first two for different values of $\alpha$ and $\beta$. We see that the 2, 4 and 6 hour period instances perform similarly when it comes to reschedule percentage, with the 8 and 12 hour periods performing slightly worse. When it comes to alternative tray percentages, the two hour period at points performs worse than even the 24 hour period for similar inventory levels, while the other periods all seem to perform similarly. Given that the median tray sterilization time is 3 hours and 18 minutes, it is no surprise that methods with periods that match that closely generally perform well, since they most closely conform to reality.

**Fig. 5:**
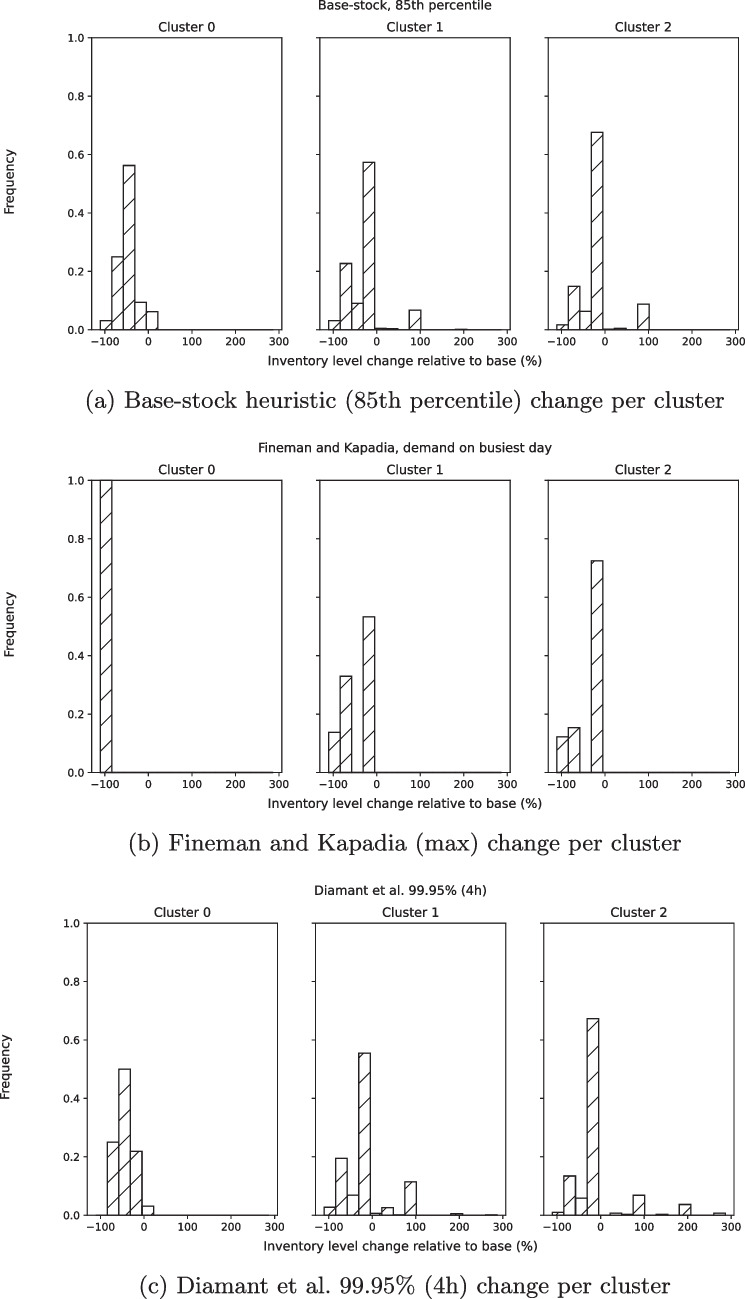
Relative inventory change per cluster

A key question is how the heuristics change the inventory per tray type. As it is infeasible to display the inventory level per tray type for all 1213 tray types for each of the heuristics, we summarized the results per tray cluster. Figure 5 displays the relative change for each cluster for one instance each for all three heuristics. Each graph displays the frequency of tray alterations (vertical axis) per alteration magnitude (horizontal axis). We observe that the alteration patterns are similar for the BS and DI heuristics. Furthermore, for these heuristics the graphs show that inventory levels can safely be lowered for many tray types in cluster 0, while maintaining similar overall performance by increasing the inventory in a relatively low (approx. 10%) number of tray types, mostly in Cluster 1 and 2.

We conclude that serious cost savings are possible by re-calibrating the tray inventory using either the BS or DI heuristics. Furthermore, we have shown that our relatively straightforward BS heuristics performs almost similar compared to the much more sophisticated and harder-to-implement DI heuristic.

### Managerial insights

The main goal of this work was to establish a method to evaluate and optimize the partnering hospital’s tray stock levels. This section shows that the BS heuristic we propose outperforms more complex ones from literature, which is an important practical insight. The implications of our study are currently being discussed with our partnering hospital’s managers. Implementation of our method is easy from a technical perspective, as it can easily be done using any spreadsheet software. Our results are not yet implemented as, due to the governance structure, a lot of stakeholders need to be convinced before such changed can be implemented. However, the promising potential savings have initiated the discussion of the optimization of the hospital’s tray inventory.

For an analytics project in healthcare to be successful it takes stakeholder engagement, technical performance, implementation and sustained use [39]. This is one of the reasons we purposely propose a simple and understandable method as this would increase the chance of actual implementation in practice. Doing so we prevent creating a so-called black box model, which is known to delay and prevent adaptation of models [40]. Based on these requirements, we formulate the following managerial recommendations for a successful implementation of our results.

- Use *hospital ERP data* to tune the simulation model and surgery generation parameters.
- Together with *all stakeholders*, which includes managers, medical doctors and CSS specialists, decide upon an acceptable reschedule percentage and alternative tray percentage.
- Use a figure similar to Fig. 5 to find the right tray inventory levels for the chosen risk level.

Following this route, hospital managers should be able to attain major savings and at the same time increased quality of care.

## Conclusion and future work

This study proposes an integrated and holistic approach to optimise the tray inventory of hospitals. We introduce a simple and easy-to-implement heuristic, which we benchmark against two well-known results from literature. Contrary to most work on this topic, we take the full complexity of the cyclic sterilisation system into consideration using a DES model, by which we can account for all sources of variability in the entire process.

The DES model constitutes the entire flow of sterile surgical trays in a hospital, and therefore takes the closed-loop nature of the flow into account. It also includes a novel method to generate surgeries and outpatient procedures, which was key to modelling the entire RMD flow comprehensively. These procedures constituted both the demand for sterile trays and the source of trays to clean, making them critical for understanding the RMD cycle. Incorporating this surgery generation procedure allowed us to comprehensively model the CSS operations and the base stock of the trays.

We apply our DES model and the proposed heuristics to a Dutch partnering hospital. We find that using our heuristic, the hospital can reduce its tray inventory by 28%, which results in significant costs savings, without compromising performance in terms of tray availability. Furthermore, we show that our easy-to-implement method performs almost as good as much more complicated methods from recent literature. We also provide managerial insights following our results, which show the usefulness of our results to other practitioners in the field.

As future work, it would be a valuable addition to the model to incorporate tray composition, thus enabling a more comprehensive analysis of the RMD flow. Additionally, there is potential in using the model to devise surgical planning guidelines aimed at reducing the likelihood of tray stock-outs. We acknowledge that the heuristics we proposed for base stock dimensioning were based on a one-rule-fits-all approach, and that developing finer heuristics tailored to the demand characteristics of each tray type might provide future researchers with a larger reduction for high-use trays and more robustness and safety for low-use ones.

## Appendix A Snapshot of the tray log dataset extracted from the ERP

**Table 10:** A snapshot of the usage log of RMDs in the hospital

Set ID	Set Name	Activity	Time	Date	Set Type ID
1	BASE TRAY	Reset	10:53:05	19-2-2020	E1
2	COLOSCOPE	On OR	10:54:44	19-2-2020	E2
3	SURG.DERMA	Wrapping Room	10:57:04	19-2-2020	E3
4	ENDO EYE 0$^{\circ }$	Wrapping Room	10:57:17	19-2-2020	E4
4	ENDO EYE 0$^{\circ }$	Reset	10:57:22	19-2-2020	E4

Source: the hospitals’ ERP system

## Appendix B Snapshot of the surgery log dataset extracted from the ERP

**Table 11:** A snapshot of the surgery log

Surg. ID	Date	Surgery Type	OR	Tray ID	Start	End
1	07/04/2021	LAPAROSCOPY DIAGN.	UH7	10	19:30	20:32
1	07/04/2021	LAPAROSCOPY DIAGN.	UH7	11	19:30	20:32
2	21/12/2021	SECTIO CAESAREA	UH6	12	19:00	19:51
3	09/07/2021	URETHROTOMY	UH8	13	18:40	19:04
4	14/03/2021	ABCES TRTMNT -(+ PCH)	UH8	14	17:23	18:17
5	28/07/2021	URETERORENOSCOPY	UH6	15	16:48	17:42
5	28/07/2021	URETERORENOSCOPY	UH6	16	16:48	17:42

Source: the hospitals’ ERP system
